# Antioxidant Activities of Phytochemicals in Fruits and Vegetables

**DOI:** 10.3390/antiox15080999

**Published:** 2026-08-12

**Authors:** Wen Qin

**Affiliations:** College of Food Science, Sichuan Agricultural University, No. 46, Xinkang Road, Ya’an 625014, China; qinwen@sicau.edu.cn; Tel./Fax: +86-83-5288-2403

## 1. Introduction

Polyphenols are a large class of secondary metabolites abundant in fruits and vegetables, renowned for their antioxidant and anti-inflammatory activities [1]. They encompass flavonoids, phenolic acids, stilbenes, lignans, and tannins, each with distinct structural features and biological actions. Dietary polyphenols can modulate the Nrf2/ARE pathway, inhibit NF-κB signalling, and interact with gut microbiota in a bidirectional manner: they promote beneficial bacteria (*Bifidobacterium*, *Lactobacillus*, Akkermansia) and reduce the *Firmicutes*/*Bacteroidetes* ratio, while gut microbes convert polyphenols into bioactive metabolites (such as urolithins, short-chain fatty acids) that enhance host antioxidant defences [2,3]. This interplay positions polyphenols as promising nutritional interventions for intestinal inflammation, particularly ulcerative colitis (UC), which affects about 5 million people globally [4]. The six original articles collected in this Special Issue illustrate the remarkable diversity of antioxidant phytochemicals from fruits and vegetables, ranging from phenanthrenes in yam peel and carotenoids in *Rubus* fruits to anthocyanins in blueberries and polyphenols in artichoke and fig wastes [5,6,7,8,9,10]. The articles collectively reinforce the central theme that dietary plant compounds offer multifaceted strategies for managing oxidative stress and inflammation.

## 2. Classification and General Mechanisms

**Figure 1:**
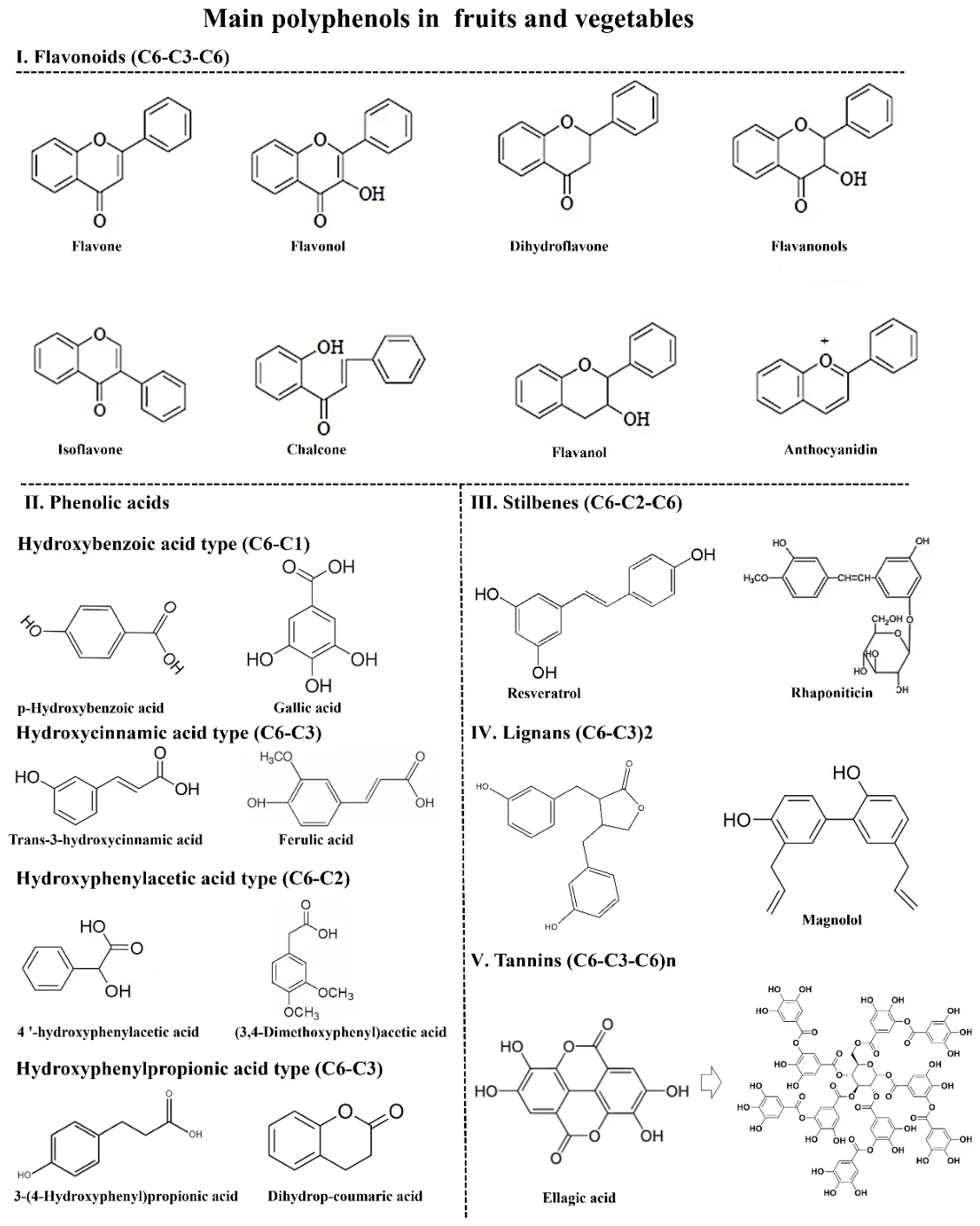
Classification and structure of different polyphenol subtypes in fruits and vegetables.

Flavonoids (such as anthocyanins and flavonols) feature a C6-C3-C6 skeleton and inhibit intestinal inflammation by strengthening tight junctions, scavenging ROS, and blocking NF-κB and MAPK pathways [10,11]. Phenolic acids (hydroxybenzoic and hydroxycinnamic derivatives) suppress NF-κB/MAPK, enhance barrier proteins, and modulate microbiota [12]. Stilbenes (such as resveratrol) activate SIRT1/Nrf2, inhibit COX-2, and reinforce epithelial integrity [13]. Lignans are metabolised by gut microbiota into enterolignans that modulate NF-κB and cytokine production [14]. Tannins (hydrolysable and condensed) exhibit strong metal-chelating and protein-precipitating properties, alleviating colitis via AMPK/NF-κB/STAT3 pathway inhibition and microbial modulation [15]. Despite these shared mechanisms, each subclass shows compound-specific efficacy, which is influenced by hydroxylation patterns, glycosylation, and molecular weight (Figure 1).

## 3. Effects and Mechanisms in Intestinal Inflammation

### 3.1. Flavonoids

Flavonoids like cyanidin-3-O-glucoside (C3G) and dihydroquercetin attenuate DSS-induced colitis by restoring microbial diversity, increasing SCFA production, and inhibiting the PI3K-Akt pathway [16]. Epigallocatechin gallate reduces inflammatory cytokines in a microbiota-dependent manner [17]. Icariin suppresses Th1/Th17 differentiation, while corylin modulates the gut–brain axis [18,19]. Barrier protection is achieved through the upregulation of ZO-1 and occludin via NADPH oxidase downregulation. Importantly, the work of Ma et al. [7] in this Special Issue demonstrates that C3G also exerts neuroprotective effects against amyloid-β-induced apoptosis by regulating Ca^2+^ homeostasis and mitochondrial function, underscoring the multi-target potential of flavonoids beyond the gut.

### 3.2. Phenolic Acids

Gallic acid, chlorogenic acid (CGA), and protocatechuic acid remodel the microbiota by enriching *Lactobacillus* and *Akkermansia* while suppressing pathogenic *Enterobacteriaceae* [20,21,22]. They restore goblet cells and MUC2 expression, and suppress caspase-dependent apoptosis via TXNIP/NLRP3 inhibition [23]. Quinic acid dually targets TLR4/NF-κB and iNOS/NO pathways [24,25]. The Special Issue article by Laghezza Masci et al. [8] shows that artichoke by-product extracts, rich in phenolic acids, reduce ROS and restore GSH in neuronal cells and improve survival in *Drosophila*, highlighting the broader antioxidant capacity of these compounds.

### 3.3. Stilbenes

Resveratrol and pterostilbene enrich *Akkermansia* and *Bacteroides*, activate Nrf2/HO-1, and enhance MUC2 retention [25,26]. However, their therapeutic window is narrow; high doses may exert pro-oxidant effects. The extraction of polyphenols from discarded fig fruits using deep eutectic solvents (DESs), as reported by Zhang et al. [9] in this issue, provides a green approach to obtain stilbene-rich extracts with high antioxidant and enzyme-inhibitory activities, offering a sustainable source for future anti-inflammatory applications.

### 3.4. Lignans

Lignans such as secoisolariciresinol diglucoside (SDG) and sesamin inhibit NF-κB and MAPK, reduce IL-6 and TNF-α, and prevent gut barrier damage by enhancing occludin and claudin-1 [27,28]. Their bioactivity depends heavily on microbial conversion; germ-free animals show negligible effects. The carotenoid-rich *Rubus* fruits studied by Piechowiak et al. [6] in this issue, although not lignans, exemplify how regional and genetic diversity influence phytochemical profiles and antioxidant capacity, reinforcing the need for tailored dietary interventions.

### 3.5. Tannins

Persimmon-derived condensed tannins and punicalagin from pomegranate mitigate colitis by modulating *Bacteroides* populations, suppressing leukocyte trafficking, and activating AMPK/NF-κB/STAT3 signalling [29,30]. Microbial metabolites like urolithin A enhance barrier integrity via AhR-Nrf2-mediated tight junction upregulation. The Special Issue also features the work of Kim et al. [5] on yam peel phenanthrenes, which, though focused on skin protection, demonstrates that tannin-like polyphenols from waste streams possess potent antioxidant and MMP-inhibitory activities, suggesting their potential in inflammatory conditions beyond the intestine.

## 4. Strategies to Improve Bioavailability

**Figure 2:**
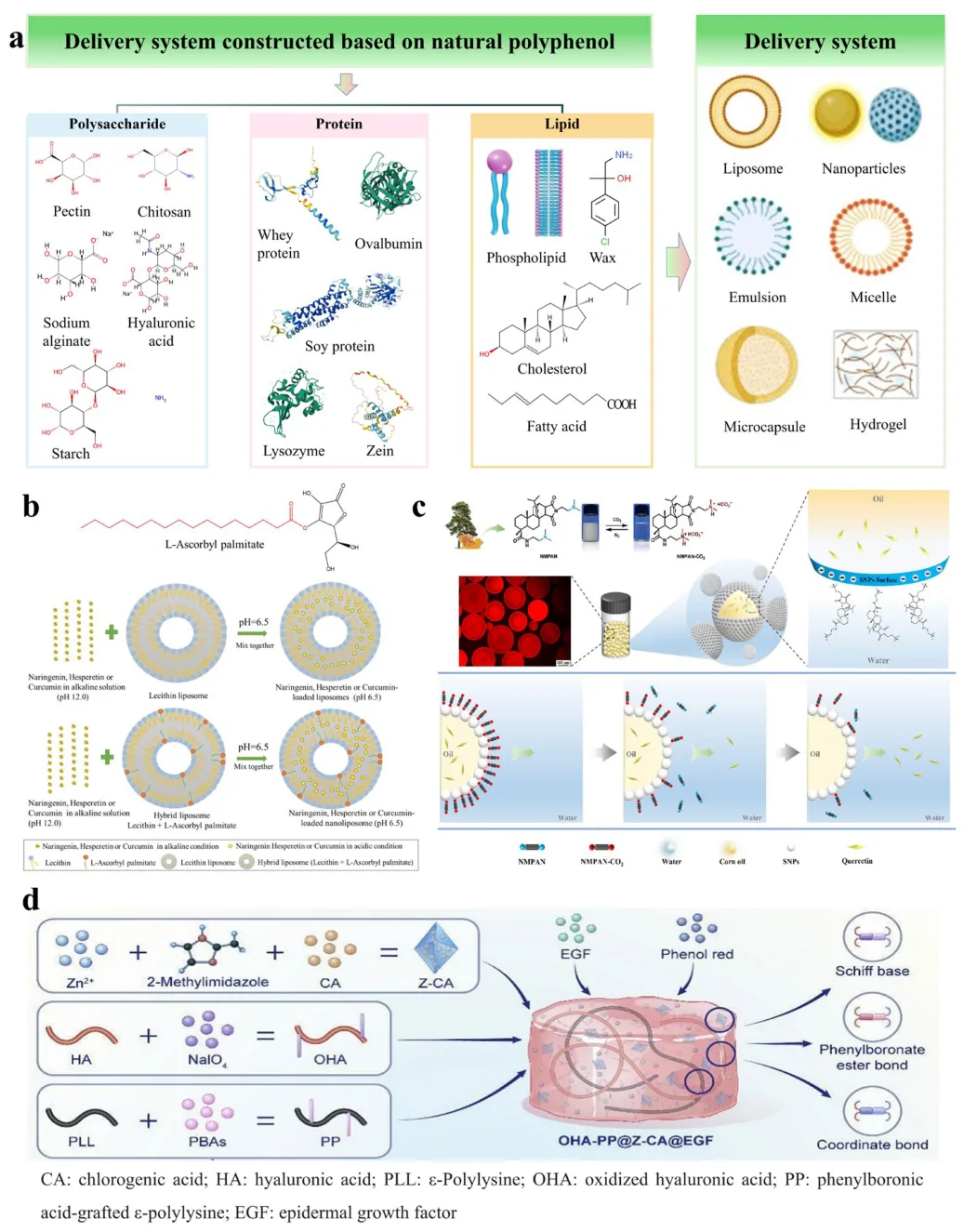
(**a**) Delivery systems constructed based on natural polyphenols; (**b**) molecular structure of l-ascorbyl palmitate and schematic illustration of the formation mechanism of naringenin, hesperetin, and curcumin loaded into a pure LAP hybrid liposome via a pH-driven method [31]; (**c**) quercetin Pickering emulsion; (**d**) chlorogenic acid-loaded nanocomposite hydrogel [41].

Poor solubility, rapid metabolism, and instability in the GI tract limit polyphenol efficacy. Several emerging technologies address these challenges (Figure 2).

Delivery systems: Nano-liposomes, Pickering emulsions, and protein/polysaccharide complexes enhance solubility and enable targeted colonic release [31,32]. Curcumin-loaded high internal phase emulsions have shown superior anti-colitis effects [33].

Structure engineering: Covalent conjugation with proteins (such as pea 7S protein-C3G) or polysaccharides (quercetin–carboxymethyl starch) improves digestive stability by >30% [34,35].

Microbiota modulation: Co-administration with probiotics (*Lactobacillus*, *Bifidobacterium*) enhances polyphenol biotransformation; for instance, *B. animalis* protects proanthocyanidins during digestion [36,37].

Processing optimisation: Non-thermal techniques (ultrasound, pulsed electric fields) and green solvents (DESs) increase bio-accessibility without degrading bioactive. The work by Zhang et al. [9] on the DES extraction of fig polyphenols and by Moura et al. [10,11] on the advanced processing of pearl millet grains exemplify how innovative technologies can preserve or even enhance antioxidant quality while reducing antinutritional factors.

Synthetic biology and precision nutrition: Engineered *E. coli* and *Saccharomyces cerevisiae* can produce high yields of flavonoids and stilbenes [38,39]. Personalised intake plans based on genomic and metabolomic data, combined with probiotic ratios, optimise utilisation for low absorbers [40].

## 5. Innovation Potential and Safety Considerations

Polyphenols offer unique advantages over conventional immunosuppressants: they act as prebiotics, reinforce the epithelial barrier, and modulate multiple inflammatory pathways simultaneously. Nanoformulations and synergistic combinations with polysaccharides (such as *Ganoderma lucidum*) are being explored for functional foods tailored to IBD management [40,42,43]. However, high doses may provoke pro-oxidant effects, gastrointestinal irritation, or reproductive toxicity [44]. Resveratrol at ≥1000 mg/day can cause diarrhoea, while genistein and curcumin have shown negative effects on sperm motility and embryonic development in animal models [45,46]. Therefore, dose optimisation and long-term safety studies are essential. The six Special Issue articles collectively emphasise the importance of characterising both the bioactivity and potential toxicity of phytochemical extracts from various sources [5,6,7,8,9,10].

## 6. Conclusions and Future Perspectives

Polyphenols are versatile natural agents that mitigate intestinal inflammation through direct antioxidant actions, the modulation of gut microbiota, and the regulation of host signalling pathways (NF-κB, Nrf2, AMPK). Their structural diversity dictates specific mechanisms, yet common challenges such as low bioavailability and rapid metabolism can be overcome by advanced delivery systems, enzymatic modifications, green processing, and personalised probiotic combinations. The research presented in this Special Issue, from the valorisation of yam peel and artichoke wastes to the neuroprotective effects of C3G and the characterisation of carotenoids in *Rubus* fruits, illustrates the breadth of phytochemical activities and underscores the importance of sustainable sourcing and innovative technologies. Future work should prioritise well-designed clinical trials, integrate multi-omics approaches to identify responder profiles, and develop regulatory frameworks for standardised polyphenol-based functional foods. By bridging traditional natural product chemistry with cutting-edge biotechnology, polyphenols hold promise for the safer, multi-targeted management of inflammatory bowel diseases and beyond.

## Data Availability

No new data were created or analyzed in this study. Data sharing is not applicable to this article.
